# The Gating Mechanism of the Human Aquaporin 5 Revealed by Molecular Dynamics Simulations

**DOI:** 10.1371/journal.pone.0059897

**Published:** 2013-04-02

**Authors:** Lorant Janosi, Matteo Ceccarelli

**Affiliations:** Department of Physics, University of Cagliari, Cagliari, Italy; University of Leeds, United Kingdom

## Abstract

Aquaporins are protein channels located across the cell membrane with the role of conducting water or other small sugar alcohol molecules (aquaglyceroporins). The high-resolution X-ray structure of the human aquaporin 5 (HsAQP5) shows that HsAQP5, as all the other known aquaporins, exhibits tetrameric structure. By means of molecular dynamics simulations we analyzed the role of spontaneous fluctuations on the structural behavior of the human AQP5. We found that different conformations within the tetramer lead to a distribution of monomeric channel structures, which can be characterized as *open* or *closed*. The switch between the two states of a channel is a tap-like mechanism at the cytoplasmic end which regulates the water passage through the pore. The channel is *closed* by a translation of the His67 residue inside the pore. Moreover, water permeation rate calculations revealed that the selectivity filter, located at the other end of the channel, regulates the flow rate of water molecules when the channel is *open*, by locally modifying the orientation of His173. Furthermore, the calculated permeation rates of a fully *open* channel are in good agreement with the reported experimental value.

## Introduction

One of the fundamental physiological functions of a cell is the molecular transport across cellular membrane. However, many of these molecules don’t permeate the membrane or they do it at a rate that is too small to be have any physiological relevance. Therefore, cells use highly selective transmembrane (TM) protein channels to allow and control molecular transport across their boundaries.

The fastest transporters are the water and ion channels, which allow passive passage of 10^9^ waters and 10^8^ ions every second, respectively. Hence, these channels were the ideal candidates for initiating about a decade ago the *in silico* studies that can reveal their underlying protein structure-function relationship. The significant increase in computer power currently allows us to study water and ion channels using molecular dynamics (MD) simulations up to 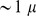
s. The premises for such investigations are the high-resolution atomic structure of the channels and accurate force fields to describe molecular interactions at atomic level. Extensive efforts to obtain better atomistic MD force fields in general [1], [2], and for ionic interactions in particular [3] have overcome many former protein channel modeling challenges, like the strong polarization effects on the environment [4], [5]. The considerably larger simulation timescales attainable nowadays enhanced our understanding of transporters, known gated systems, such as the voltage- or ligand-gated channels [6], but also lead to new findings, such as the presence of gating mechanisms in some of the aquaporin channels as well [7].

**Figure 1:**
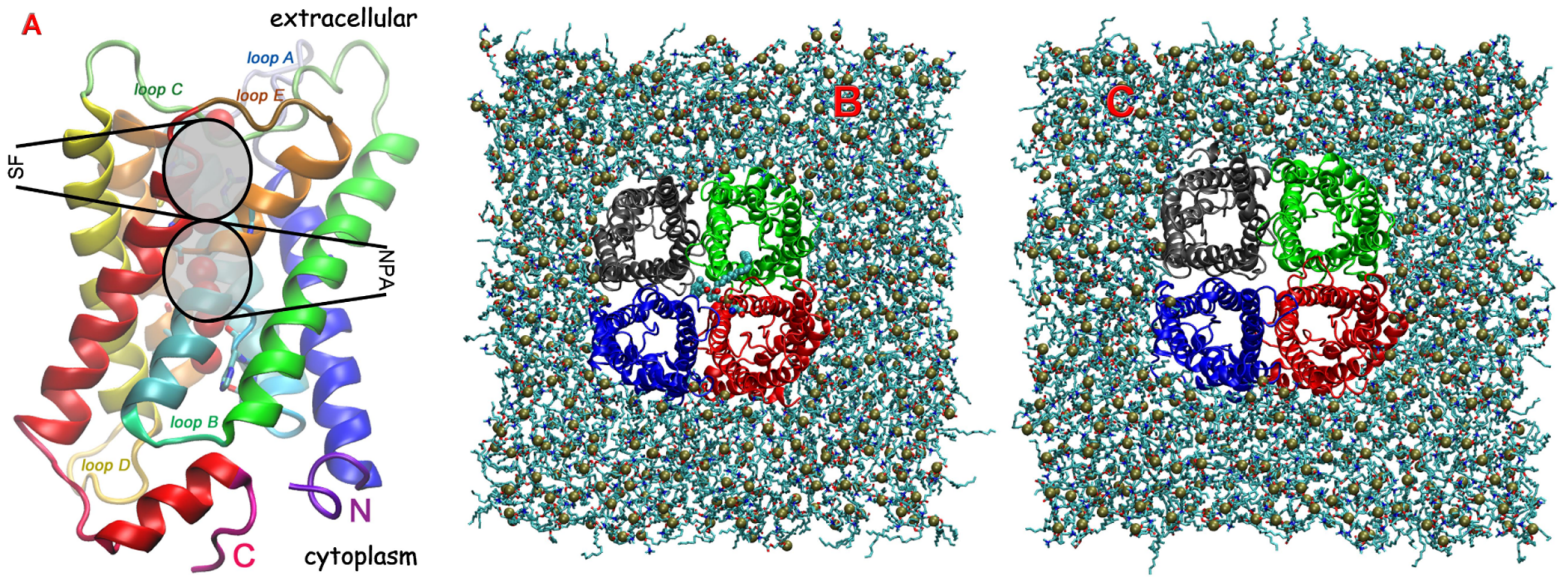
Structure of human AQP5 and the simulated tetrameric systems. (A) Tertiary structure of the crystallographic HsAQP5 (PDB databank code 3D9S) [22]. Secondary structure consists of six plus two halves of transmembrane helices connected by loops (all colored differently). Crystallographic water oxygens are shown as red balls, while important residues in the ar/R constricted selectivity filter (SF) and NPA motif regions – highlighted in darker ellipses – are shown in licorice representation, with carbons in cyan, oxygens in red, and nitrogens in blue. Panels (B) and (C) show a top view of the fully equilibrated tetramers with and without the central POPS lipid, respectively. Each channel of the tetramer is shown in a different color. Lipids are shown in line representation, with phosphorus atoms as tan balls. The central lipid is shown in ball representation with atoms colored the same as in (A). Water molecules and hydrogen atoms were removed for clarity. All renderings were done in VMD [69].

In this paper, our focus will be directed on aquaporins (see Figure 1), which is a family of transmembrane proteins with the role of conducting across the cell membrane water or additionally small sugar alcohol molecules like glycerol (aquaglyceroporins). While these molecules pass at almost diffusion rates, the pores block the passage of protons across the membrane [8]–[10]. This family of proteins performs a wide variety of physiological roles, from protecting against osmotic shock and freezing [11] to urinary concentration mechanism in kidneys [12], maintaining the eye lens transparency [13], fat metabolism, neural excitability, brain swelling, skin hydration and even cell migration (see review [14]).

Since their discovery in red blood cell membranes [15]–[17], aquaporins were found abundantly present in all kingdoms of life, from bacteria and archae, to plants, insects and mammals (see reviews [18], [19]). Thus far, 13 human aquaporins have been identified and named AQP0-12. All of the 41 aquaporins deposited in the PDB databank, of which only five human (AQP1 [9], [20], [21], AQP5 [22] and AQP4 [23]), were found to associate into tetramers (see Figure 1B,C). The human aquaporin 5 (HsAQP5), mainly localized in cells proximal to air-interacting surfaces – eyes, lachrymal, salivary and sweat glands, lungs, parts of the digestive system, such as the stomach and pancreas, but also in ovarian tumors [24]–[39] –, was the first structure crystallized in its full tetrameric organization [22]. Aquaporin structures show that these water channel proteins fold into six transmembrane (TM) 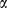-helices plus two halves. Because these halves do not cross the cell membrane, the C and N termini are both on the same side of the lipid bilayer: the cytoplasm. The high selectivity of the pores is given by two features highly conserved throughout the aquaporin family: the ar/R constriction region or selectivity filter (SF), assured by an aromatic residue and an arginine to allow passage of only very small molecules coming from the extracellular part into the pore, and the NPA (aspargine-proline-alanine) motif, which provides a high electrostatic barrier that excludes all ion leaks, including protons across the bilayer [8], [10], [40]–[42] (see Figure 1).

Recently, a gating mechanism that completely blocks the water flux at the cytoplasmic end (CE) of the pore has been reported for plant and yeast aquaporins [7], [43]–[45]. The closing mechanism is usually caused by displacement of an aquaporin-specific key residue [45]. Another mechanism type was found in amoeba, based on networks of contractile vacuoles [46]. Evidence shows that such mechanisms can be regulated by phosphorylation [7], [47], pH [48], divalent cations [49], membrane surface tension [7], [50] or high concentrations of protons, pyrophosphate and calcium [46].

Since the first simulations performed on proteins, though using short times and a very simple interaction potential, a dynamical aspect of these systems emerged [51]. For many years researchers tried to correlate the high specificity of protein functions with the existence of proper and low frequency modes involving a large number of residues, i.e., by means of the normal mode or the principal component analysis. In this context, where the gating is controlled by single key residues, unbiased MD simulations represent the more appropriate method to analyze the detailed dynamics at the microscopic scale [52]. Despite the fact that gating is controlled by an external factor, the investigation of spontaneous fluctuations induced by temperature could help identify the internal modes of the protein coupled to this process. The occurrence of these intrinsic modes without an external perturbation probably fall in the regime of rare events and in our case the tetrameric conformation of these water channels enhances the sampling statistics four fold. In addition, the full tetrameric crystal structure of HsAQP5 gives us the best interfacial alignment between the associated monomers and therefore, makes the human aquaporin 5 the perfect candidate to investigate the gating mechanism of mammalian aquaporins.

In this paper we performed a computational study of the structural dynamics and its influence on the aquaporin gating mechanism and water transport properties by means of *unbiased* fully atomistic molecular dynamics simulations. We found that different conformations within the tetrameric oligomerization state are being sampled by each pore independently, at different points in time. Furthermore, the selectivity filter and the cytoplasmic end sample *narrow* and *closed* states, respectively. While the *closed* state of the CE completely blocks the water passage by a gating mechanism characterized by the translation of the *key residue* His67 inside the pore, similarly to previously reported gating mechanisms [45], the state of the SF regulates the water flow rate through the pore when CE is *open*. Moreover, we found that the tetrameric configuration, i.e., the protein-protein coupling, is mandatory for the correct functioning of the gating mechanism. Finally, our calculated permeation rate of a completely open channel matches well the experimental value [25].

## Model and Methods

### Building of Systems

The crystal structure of the human aquaporin 5 (HsAQP5) – under the PDB code 3D9S [22] – is the first aquaporin tetramer that was crystallized in its entirety (i.e., all four monomers were captured in a single tetrameric structure). In Figure 1A we show the crystallographic tertiary structure of one HsAQP5 monomer. The aquaporin-specific regions that have paramount importance on water transport through the channel protein – i.e., the ar/R constriction region and the NPA motif region – are highlighted. The crystal structure captured in the pore displayed here six water molecules, shown as red balls. The structure of the protein is composed of six transmembrane 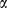-helices (each in a different color) and two halves (in cyan and orange). The helices alternatively span across the cell membrane, being interconnected by five loops: three are extracellular and two are cytoplasmic. Hence, both the C- and N-terminus are located on the cytoplasmic side of the membrane.

Although previous *in silico* studies suggested that the space in between the four channels could have a role in conducting gas molecules impermeable to that specific membrane [53], [54], this structure shows a phospholipid tail occluding the central pore. In order to assess its importance, we created model systems both with (Figure 1B) and without (Figure 1C) the central lipid. Since the crystal structure contains a lipid tail, the glycerol part of a two-tailed lipid, and the headgroup that matches best a phosphatidylserine (also suggested by Ref. [22]), our lipid model of choice was POPS (palmitoyl-oleoyl-phosphatidylserine). The two systems were embedded in fully solvated POPC (palmitoyl-oleoyl-phosphatidylcholine) lipid bilayers containing 418 lipids each (with total size of system approximately 122,000 atoms each) (systems set S1).

To investigate the importance of protein-coupling of AQP5 monomers into a tetrameric structure and its influence on structural behavior, we built four systems, each containing the initial monomeric crystal structure of the four channels, embedded into fully solvated POPC bilayers containing at least 104 lipids each (with total system size over 41,000 atoms each) (systems set S2).

### Molecular Dynamics

All simulations are fully atomistic and were carried out using NAMD 2.7 [55], CHARMM36 forcefield for lipids and proteins [1], and TIP3P for water [56], under the following protocol. A cutoff distance of 12 Å (switching function starting at 9 Å) for van der Waals interactions was assumed. Periodic boundary conditions were enabled in order to minimize the finite size effects. Long-range electrostatic interactions were computed by employing the smooth Particle Mesh Ewald (PME) method [57] with a grid spacing of approximately 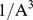. The equations of motion were integrated with a 2 fs time step with SHAKE constraint applied on all hydrogen atoms [58]. Short-range nonbonded interactions were determined at every time step, while the long-range electrostatic interactions at every two steps. Production runs were performed at a constant temperature of 310 K by employing a Langevin thermostat with a damping coefficient of 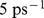. All MD simulations were conducted at constant pressure of 1 atm. by using the Nosé-Hoover Langevin piston method with a decay period of 100 fs and a damping timescale of 100 fs.

All systems were first minimized in two stages: (i) protein atoms were fixed to remove all bad contacts with water/bilayer, and (ii) then harmonically restrained to allow local relaxation. Second, the systems were gradually heated with a rate of 1 K/ps until they reached 310 K, with the heavy atoms of the tetramer still restrained. At the end of the process the lipids appeared uniformly distributed around the tetramer. Finally, the systems were subjected to long equilibration runs at 310 K and normal pressure as follows: sets S1 for 200 ns each, and S2 for 100 ns each, summing up to a total of 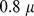
s.

## Results and Discussion

The main goal of our study is to characterize the structures of individual aquaporin channels within their natural tetrameric association. Such analysis can be done using several order parameters, such as the location and conformation of aminoacids, secondary structure of the protein etc. However, a first approach to find more general structural differences and even categorize them is to use relatively simple order parameters. Our analysis will focus on order parameters (OPs) which are good local constriction indicators (LCIs) and orthogonal to channel axis, such as distances across these sections of the pore.

For better understanding of the gating mechanism in the HsAQP5 we will focus on the regions that might play a key role in physically blocking water conduction through the channel protein: the extracellular end of the channel, i.e., the ar/R selectivity filter (SF), and the cytoplasmic end (CE). The latter has been found to present a structural gating mechanism in mammalian AQP0, plant SoPIP2;1 and yeast AQY1 aquaporins [7], [59]–[61]. In all three cases one residue displaces inside the pore, interrupting the water file.

### Selectivity Filter Exhibits Wide/Narrow Conformations

**Figure 2:**
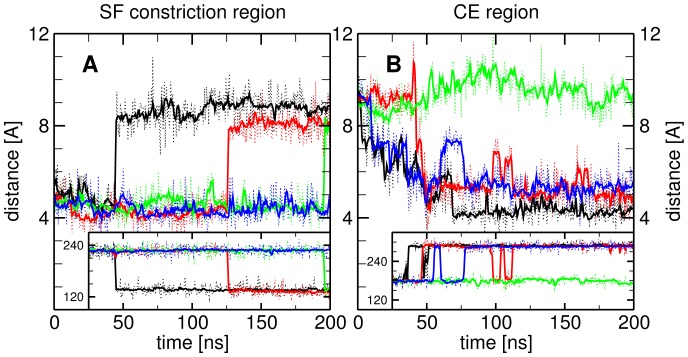
Order parameters characterizing the states of the selectivity filter (SF) and of the cytoplasmic end (CE). (A) Evolution of D1 order parameter (distance between 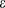-nitrogen (NE2) of His173 ring and backbone oxygen (O) of Ser183) show transitions between *wide* and *narrow* states in the selectivity filter, which are mainly caused by the His173’s ring reorientation (see inset). (B) D2 order parameter (distance between the 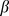-carbon (CB) of His67 and CD carbon of Ile165) shows that CE region can be characterized by *open* (green channel) and *closed* (the other channels) states. Transitions to intermediate states of D2 (6.5 Å to 7.5 Å) are correlated with His67’s sidechain orientation (see inset).

While the conserved arginine Arg188 is a major contributor to the creation of the constriction in the ar/R region, we found its orientation and position quite robust throughout the HsAQP5 simulations. However, an excellent LCI we found for the SF is the distance between the NE2 nitrogen atom of the His173 ring and the backbone oxygen atom of Ser183 (hereafter denoted as D1), which is greater than the SF diameter. Figure 2A shows clearly the presence of two states. In the first one, when 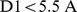, His173 and Ser183 are very close to each other and restrict the water passage in the ar/R constriction region: the SF is in the *narrow* conformation. The second state, corresponding to 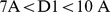, is the *wide* conformation of the SF region, which allows passage of water molecules. Furthermore, we found a direct correlation between the proximity of His173 to Ser183 and the former’s ring orientation (see the inset of Fig. 2A). Hence, when the SF switches from *narrow* to *wide* state, the His173 ring flips into its mirror orientation, i.e., the CA–CG–CD2–NE2 dihedral angle changes from an average 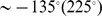 to 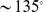, explaining the large increase in the value of D1.

**Figure 3:**
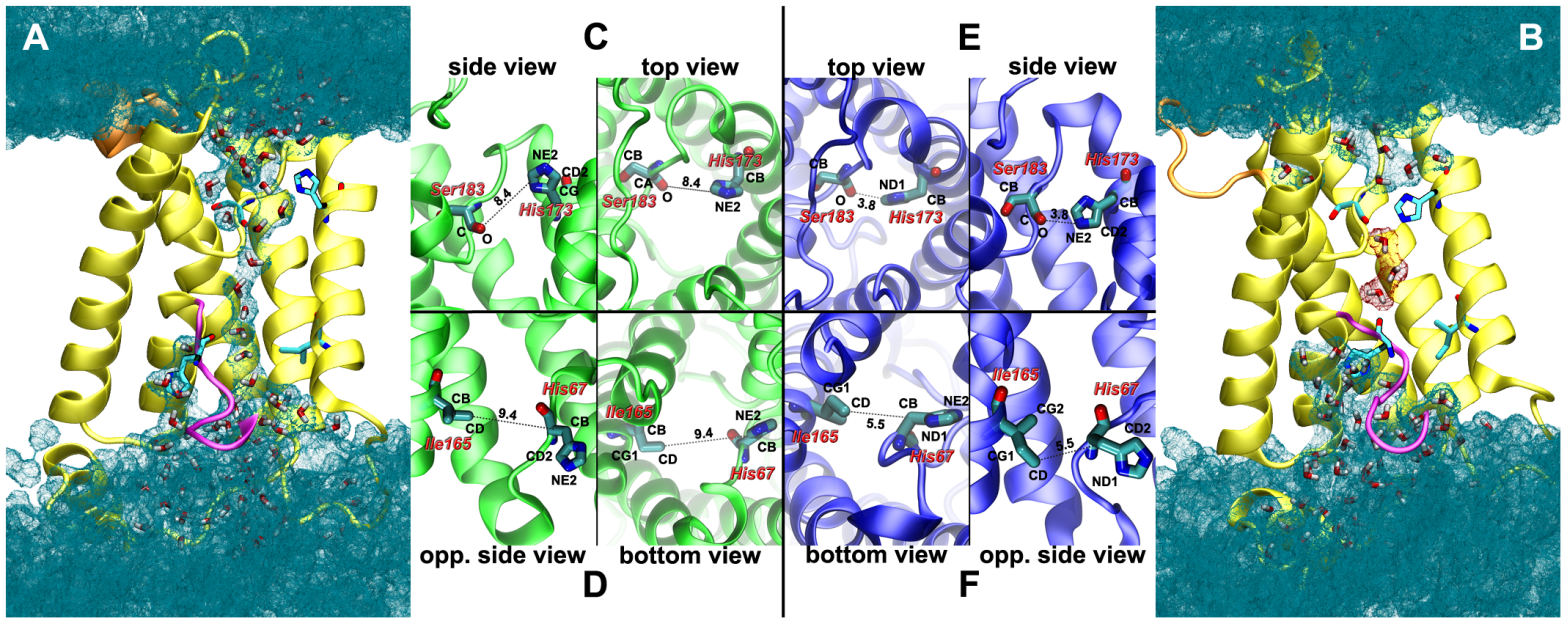
Structural view of the states of the SF and CE regions. Side view sections of (A) structure having SF and CE regions in the *wide* and *open* states (i.e., water-conducting), and of (B) structure with SF and CE regions in the *narrow* and *closed* states. Cyan wireframe surfaces show spaces occupied by water, with explicit molecular models (oxygen in red and hydrogen in white) near and inside the channel. For clarity protein is shown in yellow cartoon representation. In orange and magenta are shown the loops that go through major changes on the extracellular side and CE region, respectively (see details in the text). Residues used in order parameter definitions in the SF (His173, Ser183) and CE (His67, Ile165) regions are shown in licorice representation. Zoom on the SF region of *wide* (C) and *narrow* (E) states, and on the CE region of the *open* (D) and *closed* (F) states. Representations are kept the same as in (A) and (B). For clarity, waters were removed and protein color is the same as in previous plots. All renderings were done in VMD [69].

Figure 3 shows a close-up look at the atomic structure of the *wide* (Fig. 3C) and *narrow* (Fig. 3E) states of the ar/R constriction region. The large value of D1 in the *wide* state is due both to the sidechain orientation of His173 along the channel axis and to the ring orientation such that the NE2 atom is localized farther than other atoms of the ring. D1 was however chosen over other distances mainly due to its stability in value and clear marker of the two states.

The SF switches to the *narrow* state when the His173 sidechain changes to an orientation normal to the pore axis. This movement of the ring, which imposes stronger channel constriction, is also accompanied by a flip of the ring into its mirror position, as shown in Figure 3. It is the two conformations of the His173 that determine the two states (*wide*/*narrow*) present in the selectivity filter.

### Cytoplasmic End Exhibits Open/Closed States

The distance between the CB atom of His67 and the CD atom of Ile165 (hereafter denoted as D2), also larger than the local diameter, was used as our LCI in the CE region. Figure 2B indicates that initially (i.e., in the crystal structure) the value of D2 is relatively large in all four channels, but later on falls into two main states: *open*, with 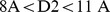, and *closed*, with 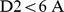, in which water passage is not allowed. Another conformation is rarely populated at D2 values between 6.5 Å and 7.5 Å. However, it seems rather unstable and most likely plays the role of being an intermediate for transitions from *open* to *closed* state or viceversa. These intermediate states are directly related to the His67 sidechain orientation (see inset of Fig. 2B), as follows. In the *closed* state the CG–CB–CA–C dihedral angle averages around 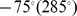. The change of this angle, describing the aminoacid sidechain orientation, is correlated with having D2 in the intermediate state.

The *open* state in the CE region (see Figure 3D) is characterized by orientation of the CA–CB and CB–CG (which is along the histidine ring itself) bonds of His67 as almost perpendicular and parallel to the channel axis, respectively. At the same time the plane formed by the CD–CG1–CG2 atoms of the Ile165 sidechain atoms (P1) is perpendicular to the pore axis.

The *closed* state is characterized by a shift towards the pore interior of the coil connecting the second transmembrane helix (not shown in Figs. 3A,B for clarity) to the first half helix. This change is followed by a rotation of the CA–CB bond in the plane perpendicular to the channel axis and a mirror-flip of the histidine ring yielding CB–CG bond quasi-perpendicular to the pore axis. The conformational changes occur such that the CB atom moves towards the interior of the channel and the His67 ring to the side/wall of the pore. Most of the time the reorientation of His67 is followed by a flip of the P1 plane to an orientation parallel to the channel axis. While the latter does not influence the constriction of the channel, the drastic change in the positioning and orientation of His67 does, as reflected by D2.

While flipping of the His67 ring orientation correlated only once to a transition from *closed* to intermediate state of the CE region, it seriously impacts the local conformation, and therefore could potentially play a role in *closed* to *open* transition or viceversa. The local structural change consists in the formation of a water pocket parallel to the main conducting pore of the channel, The pocket is directly linked to the bulk water on the cytoplasmic side of the membrane (see Figs. 3A,B).

Moreover, this pocket allows the formation of water hydrogen-bond (H-bond) chains up to Ile68, which in the *open* state has no water in the vicinity. While the pore is open (no side pocket), the backbone oxygens of Gly65, Gly66 and His67 each form with different waters hydrogen bonds perpendicular to the channel axis. As the coil shifts inside the pore, Gly66’s oxygen moves, on average, from 5.8 Å to 3.6 Å from the Ile165’s CD atom. This shift is a tap-like constriction move that breaks the single file water H-bond chain (see Figs. 3A,B) and completely blocks water transport through the channel. Thus, Gly65 and His67 backbone oxygens change orientation downwards and upwards, respectively, to form H-bonds with other water molecules.

### Modulation of the States of the Selectivity Filter and Cytoplasmic End Regions

In order to shed more light onto the structural behavior we found for the human AQP5, we looked into how it is influenced by the tetrameric state of the channels and by the central lipid, found so far only in the cavity of HsAQP5 [22]. For best quantitative comparison we followed how the order parameters used to identify the *open/wide* and *closed/narrow* states in the tetrameric channels change when only single channels are simulated in the lipid bilayer and when the central lipid is removed from the tetrameric structure.

We found that both D1 and D2 states are present in the single channel simulations as well (see Figure S1), showing that the two states at both ends of a channel are to some degree intrinsic to each protein channel. Besides the fact that both D1 and D2 have larger values, suggesting the pores are wider in the absence of the tetrameric protein coupling, they also present much larger fluctuations (see Figure S1), indicating less stability in monomeric state. More importantly however, D1/D2 are mostly found in their *wide*/*open* state along the 100 ns simulations, which is quite the opposite of what we have seen in the tetrameric simulations. This suggests that the protein-protein coupling might play a crucial role in regulating the water flow through the channels, especially at the CE gating site, since D2 fluctuations in the monomers are the largest. Nonetheless, the His67 ring and sidechain orientational states are present and, as in the tetramers, they are correlated with the behavior of the CE state.

In the absence of the central lipid both D1 and D2 are greatly conserved with very little changes in their average values. Hence, when the central lipid is removed, D1 has same values in the *narrow* state and only 0.5 Å greater in the *wide* state. Similarly, D2 is 0.5 Å smaller in the *closed* state and 0.5 Å greater in the *open* state. Furthermore, the distance–dihedral correlations are also maintained. These observations can only lead to the conclusion that the central lipid has no determining role in influencing the structural behavior of the HsAQP5 channels. Hence, in order to extract the transport properties of AQP5 in various combinations of the SF and CE states, we used the simulations of the tetramer both with and without the central lipid.

### HsAQP5 Transport Properties

#### Water permeation

Finally, we investigated how the state of the channels influences their water transport properties. Specifically, we calculated the single channel diffusive (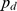) and osmotic (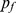) permeabilities, described in more detail in the following paragraphs. To emphasize the water permeability and occupancy differences between the *open/wide* and *closed/narrow* states of the CE and SF regions, we listed in Table 1 the values of 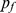, 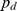, 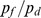 and 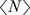 for various combinations of these states. If the water transportation occurs in ideal single-file fashion, the continuous time random walk model [62], [63] predicts that 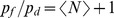, where 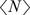 is the average number of water molecules inside the pore. While the ratio yields smaller values than expected in the conductive states, the qualitative behavior is accurate (i.e., correct relative values of permeabilities, 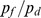 ratios and 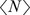). Moreover, we found the average osmotic permeability of a channel in the fully *open* state (SF is *wide* and CE is *open*) to match quite well the experimental value of 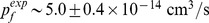 [25].

**Table 1:** Single channel osmotic and diffusive permeabilities 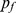 and 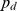 in units of 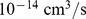 calculated over representative sections of simulations with or without central lipid that represent best the state of the SF and CE regions.

SF CE	wide open	narrow open	narrow inter	wide closed
*p_f_*	3.47	1.52	0.33	0.29
*p_d_*	0.69	0.47	0.12	0.00
*p_f_/p_d_*	5.03	3.23	2.75	–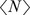	7.1±1.1	5.7±0.9	4.5±0.9	5.3±0.7

**Figure 4:**
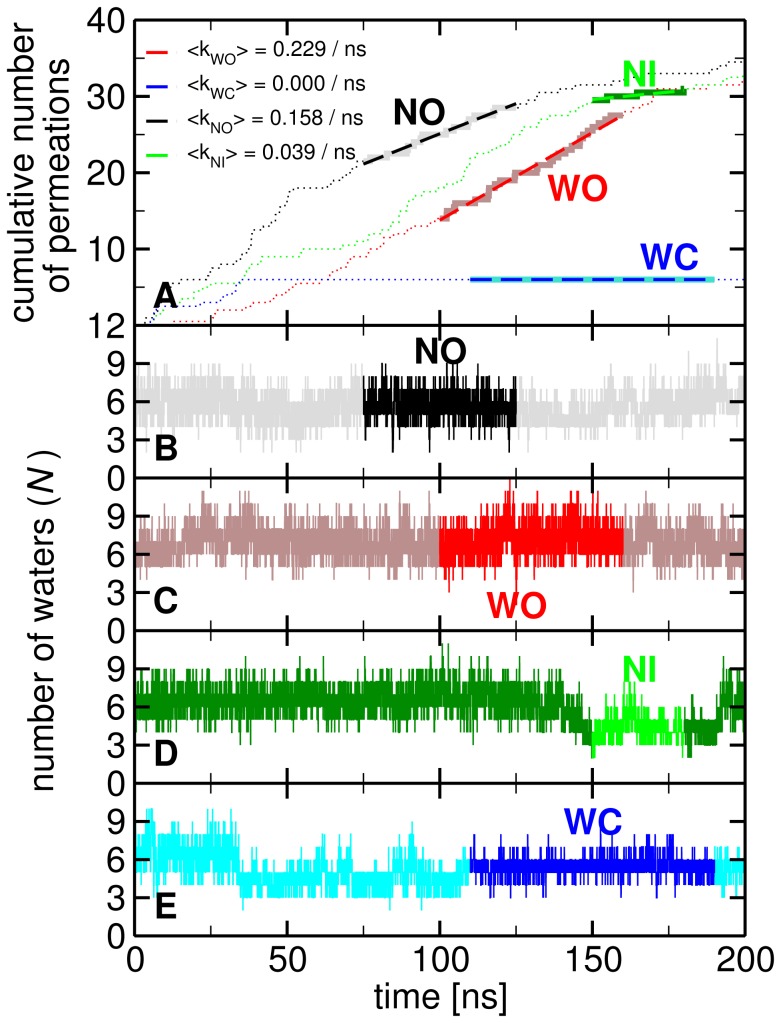
HsAQP5 water permeation and population. (A) Cumulative number of water molecule permeations as a function of time through each channel (averaged over positive and negative permeations). The regions highlighted with thick lines represent the time periods of interest, specified by the two-letter code. The first letter indicates the status of the SF (W - *wide*, N - *narrow*), while the second that of the CE region (O - *open*, C - *closed*). These portions were fit by linear functions to extract the permeation rates, shown in the legend. (B) – (E) As in panel (A), we highlighted the regions of interest and used them to extract the average number of waters in a channel in that specific state 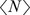. These averages are listed in Table 1 of the main text.

The diffusive permeability 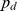 is linked to the average water permeation rate in one direction 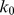 through the equation 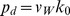 [63], where 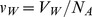 is the specific volume of a water molecule, calculated from the molar volume of water 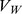 (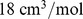) and Avogadro’s number 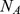. Cumulative permeation rates clearly show the highest permeation rate for channels *open* in the CE region and very low/zero permeation rate for intermediate/*closed* state (see Figure 4A). In the SF water permeates even when in *narrow* state, although at much lower rates as compared to the *wide* conformation (details below).

The osmotic permeability 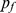 can be estimated using a linear regression of the mean square displacement of the dimensionless collective variable describing the movement of all waters inside the channel 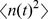 [64], [65], when *t* is much larger than the autocorrelation function of *n*. We used 2 ns windows to calculate the mean square displacement of 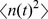 and then extract the 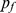 values. In Figure 4B–E we show the time evolution of the number of water molecules inside the pores *N*.

#### Channel radius profiles

**Figure 5:**
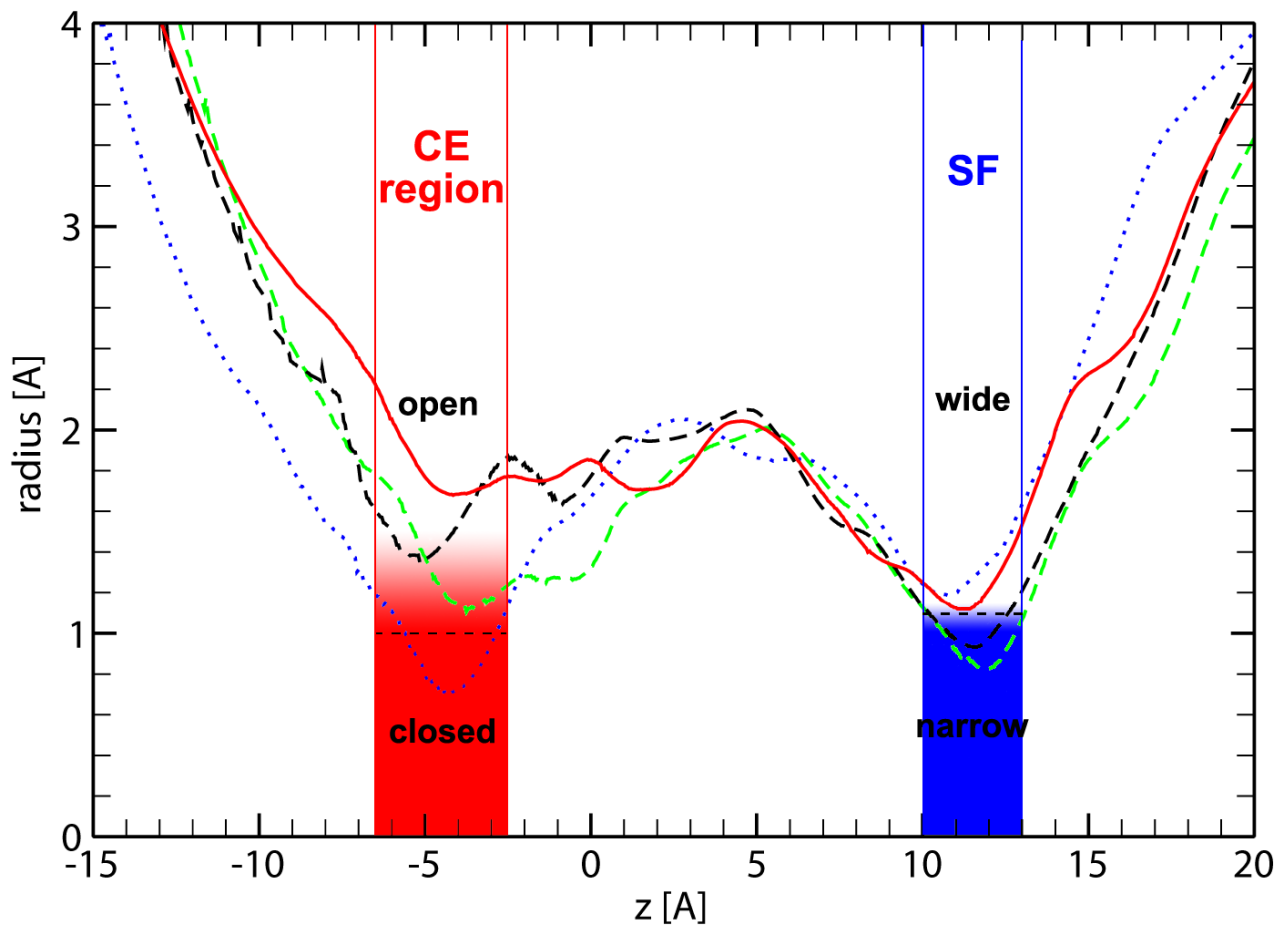
HsAQP5 pore radius profiles. Comparison emphasizing the state of the cytoplasmic side gate (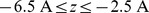): *closed* (dotted line), intermediate (dashed lines) and *open* (solid). In the SF region (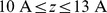) for radii greater than 1.1 Å the pore is in the *wide* state. Below, the pore corresponds to the *narrow* state.

Since water was found to permeate in states that are intermediate to the *open* and *closed* states in the CE region (see Figure 2), we calculated average channel radius profiles on *open*, intermediate and *closed* states using the program HOLE [66]. The radii were computed in the membrane plane (perpendicular to the z-axis) and are shown in Figure 5.

The four representative profiles basically show two states in the SF region: *wide* above 1.1 Å (dotted and solid lines), and *narrow* below it. In the CE region the pore is *closed* when the radius is less than 1 Å (dotted line). Above 1.4 Å the pore is in the *open* state and water permeates without any restrictions. Looking at permeation rates for pores *open* in the CE region we were able to establish that the *narrow* state of SF still allows permeation rates of 25%–50% of the *wide* state value. Using the same approach we found that the rarely visited CE intermediate state permeates at about 60%–75% of the *open* state’s permeation rate. However, the *closed* state of the CE region completely blocks water permeation. Hence, as shown in Table 1, the state of the CE region dictates if the channel will allow or block the passage of water through HsAQP5.

### Comparison to Other Aquaporins

To understand the importance of our findings on the gating mechanism and investigate potential regulating methods, we compared HsAQP5 with other known structures, and performed a multiple sequence alignment to many types of aquaporins, using ClustalW version 2 algorithm [67]. Hence, we looked into the similarities and differences of HsAQP5 with respect to human aquaporins 0–10, plant SoPIP2;1 and PIP2;2, yeast AQY1 and E.coli AQPZ (Figure S2).

One important regulation method of the gating mechanism in aquaporins [7], [45], [61], [68] was found to be phosphorylation. Similarities between the 3D structure of HsAQP5 and SoPIP2;1 in the area between loop B and the N-terminus [45] make the somewhat conserved Ser83 a potential phosphorylation site. But its relatively distant location from the gate and lack of correlation with the *open*-to-*closed* state switching suggest it is rather unlikely. However, Thr73 and Ser64 residues form hydrogen bonds/water bridges with the *key residue*, His67, which are correlated with the state of the gate. While Thr73 is sometimes replaced by a Ser (see Figure S2), this does not change its basic functional properties. Nonetheless, in the *open* state of the channel, water cannot reach Thr73, hence it is unlikely that phosphorylation can occur at this site. On the other hand, Ser64 is open to the bulk water at all times and changes conformation when the gate switches from *open* to the *closed* state. In fact, the intermediate step in switching from *open* to *closed* state consists in the formation of the water side pocket. This disrupts the Thr73–Gln94–Glu17–His67 H-bonding chain by creating temporary water bridges between some of these residues. (Please note that the latter three are 100% conserved residues in all the compared aquaporins, except for the His to Asn change in AQY1.) Because the fully conserved Ser64 forms water bridges with both Glu17 and His67, these disruptions change the orientation of Ser64’s backbone and shift Gln94 and Glu17 to the water pocket edge (number of waters near these residues doubles). The latter allows for His67’s ring reorientation which switches the gate to the *closed* state. In addition, the presence of small and highly flexible residues like Gly65, that is fully conserved, and Gly66, conserved or replaced by alanine, favor the gating movement by rotation of His67 sidechain.

Therefore, one can speculate on the existence of a reverse mechanism of opening the gate, induced by a potential phosphorylation of Ser64. However, during a 100 ns long continuation of the simulations with a phosphorylated Ser64 (after performing proper minimization and equilibration due to steric hindrance) we did observe three switching attempts from *closed* to *open* state of the gate, but none lasted (see Figure S3). This could have happened for couple of reasons: (i) the *closed*-to-*open* switching time of the gate might be much larger than 100 ns timescale; (ii) the phosphorylation may be much more complex than just changing size and charge or by mutation [7]; it may be accompanied by changes in the protonation state of residues within the local environment, like the His67.

Furthermore, both in HsAQP5 and AQY1 the blocking *key-residue* is an aminoacid with a ringed sidechain, i.e., His67 in HsAQP5 and Tyr31 in yeast. Tyr has been also found to be the blocking residue in bovine AQP0 [45], [59], [60]. This is not true however in plant PIP2;1 and PIP2;2, which have a highly conserved Leu in the narrowest part of the *closed* channel. Nonetheless, like in myoglobin where it controls the ligand migration between internal cavities by flipping orientation between long and bulky to short conformation [52], Leu can also act as a switching gate.

While in all four types of organisms, there is clearly a similar three-dimensional organization of the gating mechanism at the cytoplasmic end of the channel, its realization is quite different. Hence, in plant SoPIP2;1 the pore is occluded by loop D, in yeast AQY1 by the longer N-terminus (Tyr31 is an inserted aminoacid in AQY1 as compared to the sequence of HsAQP5), and in HsAQP5 by the coil connecting the second transmembrane helix to the first half helix.

### Conclusions

The major aim of this work was to characterize the structural dynamics of the human aquaporin 5 (HsAQP5) channels within their tetrameric configuration and how they influence the gating mechanism of aquaporins. Since water transport occurs on nanosecond time scale, we were able to use *unbiased* atomistic molecular dynamics simulations to follow the conformational changes of each channel. Such analysis gave us detailed insights on how water conduction occurs within the tetramer and how it is strictly determined by structural changes of each individual channel.

We found that the aquaporin channels visit different conformations independently at different points in time. Detailed analysis of the structural conformations of the pores revealed that the ar/R selectivity filter (SF) exhibits *wide* and *narrow* states, while the cytoplasmic end (CE) of the channel can sample *open* and *closed* states. This finding has paramount consequences on how water transport is achieved in each channel of the tetramer. The CE region’s *closed* state completely blocks water flow through the pore, suggesting the presence of a tap-like gating mechanism. The SF on the other hand can regulate the water flow rate when the CE is *open* by switching between *wide* and *narrow* states.

Atomic level details show that constrictions in the SF are due to the coupled translation of the His173 with flipping of its ring orientation from parallel to perpendicular to the channel axis. In the CE region a similar change in sidechain orientation of the His67 is coupled with a larger scale shift of the coil connecting the second transmembrane 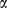-helix to the first half-helix towards the pore center, thus blocking the water flow. This structural observation was verified by osmotic permeability calculations that showed zero water permeation in the *closed* state. Moreover, permeability values of 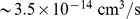 corresponding to the *open* state of the CE region match quite well the reported experimental value [25]. Our results show that the one-key-residue-based cytoplasmic gating mechanism found in other aquaporins [7], [59]–[61] is the main blocking method of water passage through the HsAQP5 as well. In this case however, the blocking *key residue* is His67.

Simulations of monomers have shown that the *wide/open* and *narrow/closed* states of the SF and CE regions are, to some degree, intrinsic to the protein. However, the tetramer simulations also showed that the channels reside in these states for many tens or even hundreds of nanoseconds. Hence, determining their population distribution needed for accurate calculation of average water transport properties can be done from free molecular dynamics simulations many microseconds long. Such time scales are still very expensive for fully atomistic models (of systems of this size), which are absolutely necessary due to the fact that water transport is single filed and that accurate local electrostatics are essential, especially in the NPA motif region [21], [40].

Finally, based on the correlation between the change of a complex network of hydrogen bonds connecting Ser64 to His67 with the opening of the CE gate, and on the corresponding serine’s participation to water regulation in yeast AQY1 by phosphorylation, we speculate that Ser64 might be the cytoplasmic end gating mechanism’s regulation by phosphorylation site for HsAQP5. While we were unable to observe such regulation on a 100 ns timescale, we hope that experimental methods will be able to confirm our hypothesis.

## Supporting Information

Figure S1 **Monomer simulations order parameters.** Time dependence of distances D1 (A) and D2 (B) (see text) in the simulations of the four independent monomers (systems set S2). Channels 1, 2, 3 and 4 are colored with the same color-coding as in the tetramer: black, red, green and blue, respectively.
(TIF)

Figure S2 **Multiple sequence alignment of HsAQP5 with other aquaporins.** ClustalW multiple sequence alignment of HsAQP5 with human aquaporins 0–10, yeast AQY1, plant SoPIP2;1 and PIP2;2, and E.coli AQPZ. ClustalX coloring was used (based on residue type and score). Top numbers show the *key residues* in switching the state of the two ends of the channel (in red) and their important interacting neighbors (in black).
(TIF)

Figure S3 **Phosphorylated Ser64 simulations CE region order parameter.** The time dependence of distance D2 (see text) in the 100 ns long extended simulation with the Ser64 phosphorylated. Only very brief attempts to switch from *closed* to *open* states are noted at 240, 280 and 295 ns, respectively.
(TIF)
