# Supplementary figures and images for: The Gating Mechanism of the Human Aquaporin 5 Revealed by Molecular Dynamics Simulations
Source: PLoS One. 2013 Apr 2;8(4):e59897. doi: 10.1371/journal.pone.0059897 (PMC3614956; doi:10.1371/journal.pone.0059897)

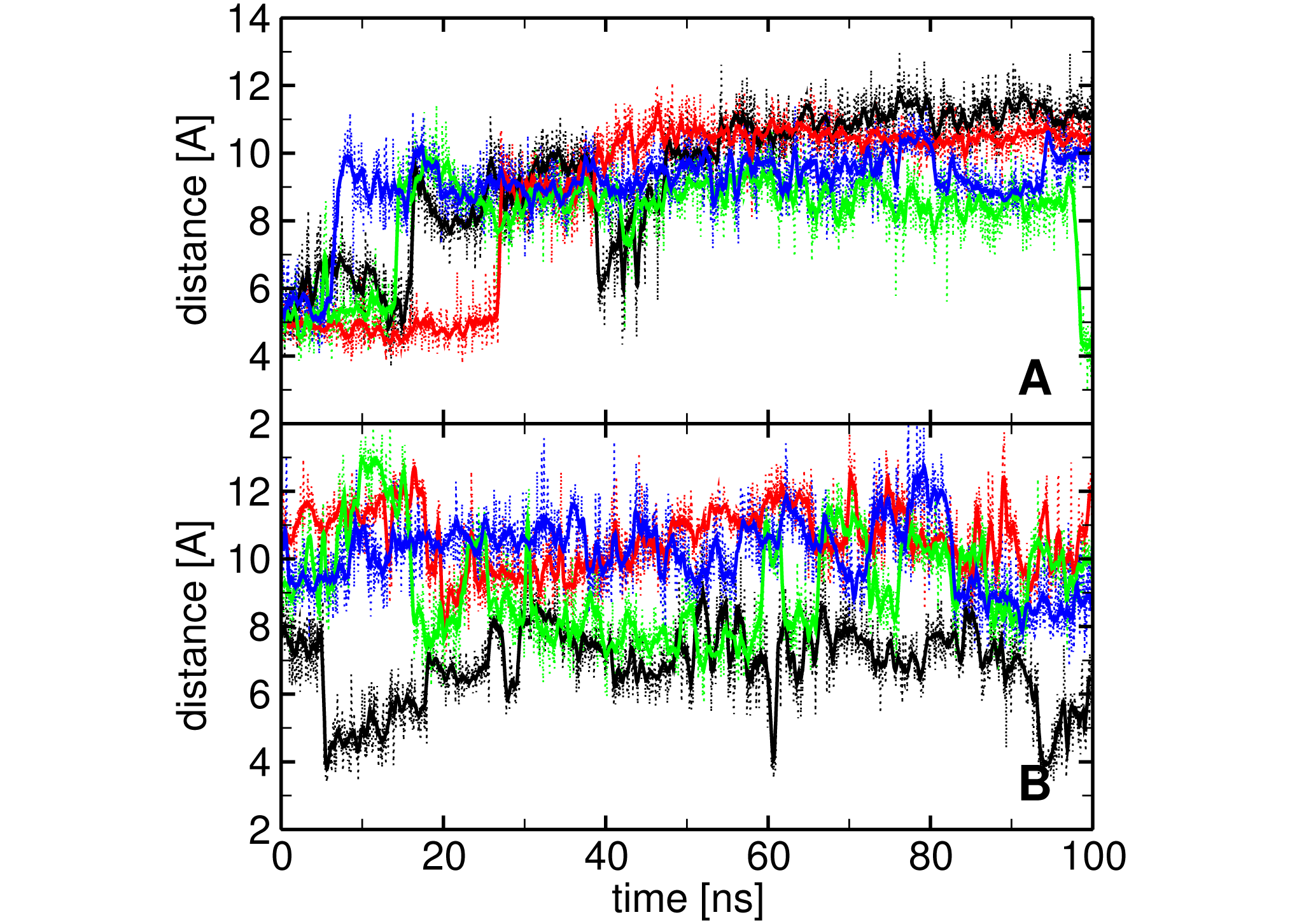

Supplement: Figure S1 — Monomer simulations order parameters. Time dependence of distances D1 (A) and D2 (B) (see text) in the simulations of the four independent monomers (systems set S2). Channels 1, 2, 3 and 4 are colored with the same color-coding as in the tetramer: black, red, green and blue, respectively. (TIF) [file pone.0059897.s001.tif]

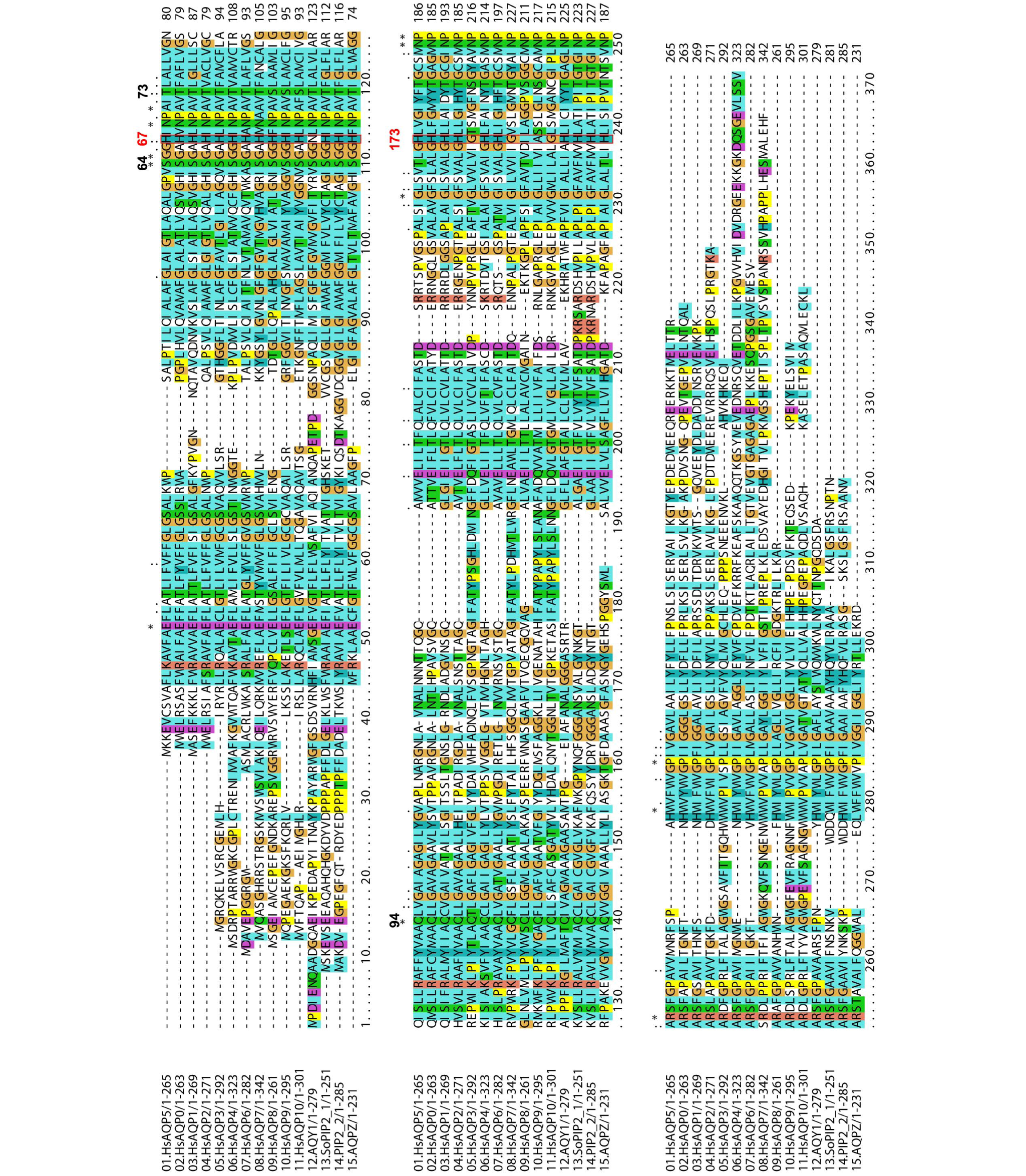

Supplement: Figure S2 — Multiple sequence alignment of HsAQP5 with other aquaporins. ClustalW multiple sequence alignment of HsAQP5 with human aquaporins 0–10, yeast AQY1, plant SoPIP2;1 and PIP2;2, and E.coli AQPZ. ClustalX coloring was used (based on residue type and score). Top numbers show the key residues in switching the state of the two ends of the channel (in red) and their important interacting neighbors (in black). (TIF) [file pone.0059897.s002.tif]

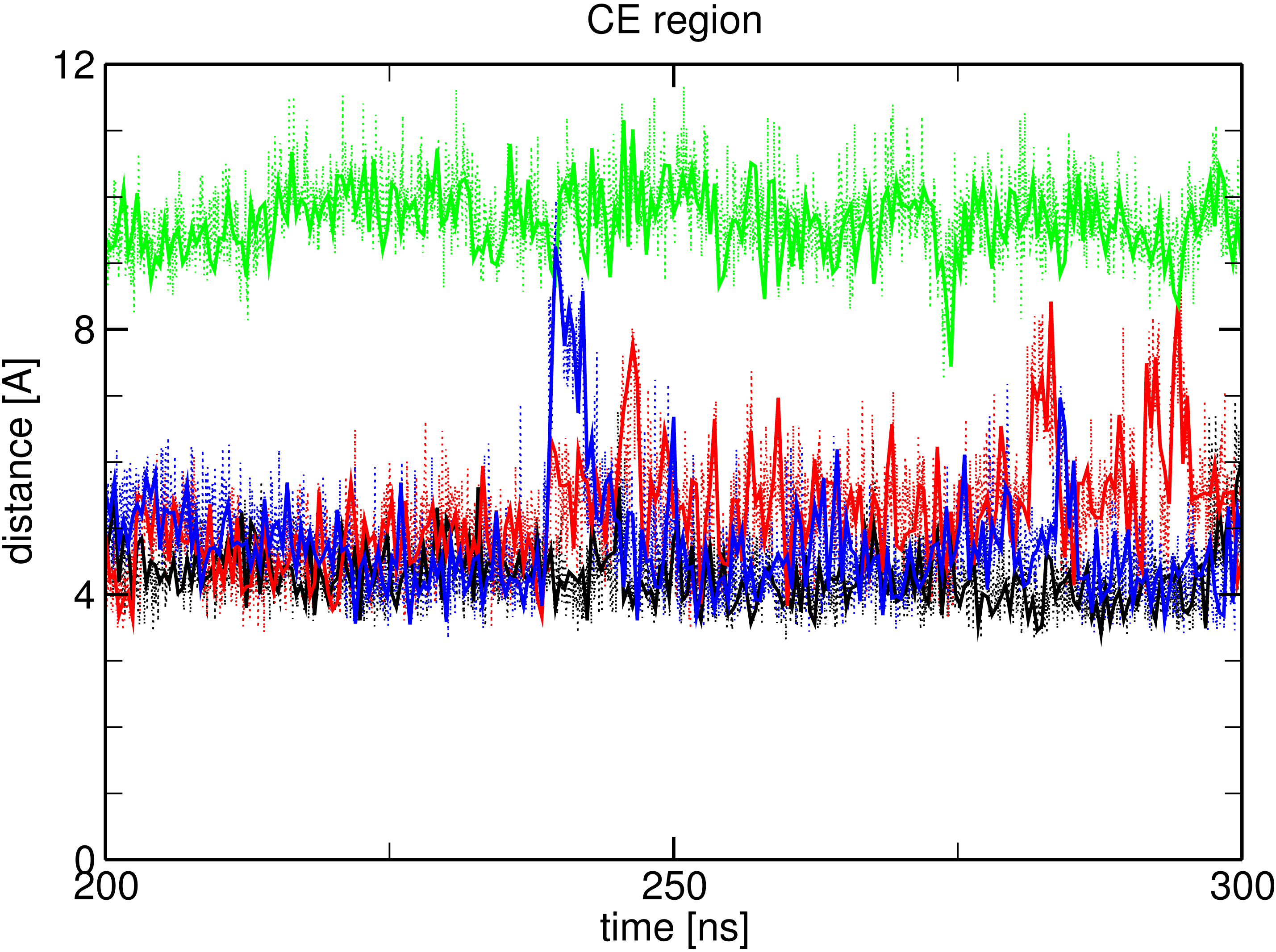

Supplement: Figure S3 — Phosphorylated Ser64 simulations CE region order parameter. The time dependence of distance D2 (see text) in the 100 ns long extended simulation with the Ser64 phosphorylated. Only very brief attempts to switch from closed to open states are noted at 240, 280 and 295 ns, respectively. (TIF) [file pone.0059897.s003.tif]
